# European principles of inhibitor management in patients with haemophilia: implications of new treatment options

**DOI:** 10.1186/s13023-020-01511-8

**Published:** 2020-08-24

**Authors:** C. Hermans, P. L. F. Giangrande, B. O’Mahony, P. de Kleijn, M. Bedford, A. Batorova, J. Blatný, K. Jansone, on behalf of the European Haemophilia Consortium (EHC) and the European Association for Haemophilia and Allied Disorders (EAHAD), J. Astermark, M. Crato, R. d’Oiron, A. Dougall, K. Fijnvandraat, S. Grønhaug, V. Jiménez-Yuste, M. Jokić, S. Lobet, B. Nolan, F. Peyvandi, A. Ryan

**Affiliations:** 1 Haemostasis and Thrombosis Unit, Division of Haematology, Cliniques Universitaires Saint-Luc, Université catholique de Louvain (UCLouvain), Brussels, Belgium; 2 European Haemophilia Consortium, Brussels, Belgium; 3 University of Oxford, Oxford, UK; 4 Trinity College, Dublin, Ireland; 5 Department of Rehabilitation, Nursing Science and Sports, University Medical Center Utrecht, Utrecht, the Netherlands; 6 Canterbury Christ Church University, Kent, UK; 7 National Hemophilia Center, Dept. of Hematology and Transfusion Medicine, School of Medicine of Comenius University and University Hospital, Bratislava, Slovakia; 8 Children’s University Hospital and Masaryk University, Brno, Czech Republic

**Keywords:** Haemophilia, Guidelines, Inhibitors, Factor VIII, Factor IX, Bypassing agents, Emicizumab, Immune tolerance

In light of the rapidly changing landscape of haemophilia treatment, the authors of the position paper on the *“*European Principles of Inhibitor Management” published in 2018 (Table 1) [1] now provide an update on the major impact of novel therapies that bypass and/or substitute clotting factor VIII (FVIII) and IX (FIX) in the care of haemophilia patients with FVIII- or FIX-neutralizing allo-inhibitory antibodies (inhibitors).

**Table 1:** European principles of inhibitor management in patients with haemophilia [1]

1. Lifelong Awareness of the Incidence of Inhibitors and Risk Factors	
2. Early Recognition and accurate diagnosis	
3. Organisation of Care and Communication Between All Stakeholders	
4. Inhibitor eradication by Immune Tolerance Induction Therapy	
5. Hemostatic Treatment with Bypassing Agents	
6. Access to and Optimal Preparation for Surgery and Invasive Procedures	
7. Delivery of Specialist Nursing Care	
8. Provision of Tailored Physiotherapy Care	
9. Access to Psychosocial Support	
10. Involvement in the Research and Innovation	

The most advanced novel agent is undoubtedly emicizumab (Hemlibra®, Roche), a bispecific antibody that mimics the function of FVIII and facilitates the coagulation cascade in haemophilia A patients both with and without inhibitors as it is not recognized by FVIII-neutralizing allo-inhibitory antibodies. Phase III clinical trials in adults (HAVEN 1) and children (HAVEN 2) have shown significant overall bleed reduction compared to prophylaxis with classical bypassing agents such as recombinant activated factor VII (rFVIIa) or activated prothrombin complex concentrate (aPCC) in patients with haemophilia A and inhibitors [2, 3]. This molecule is administered subcutaneously, once a week or less frequently, greatly alleviating the burden of intravenous injections, especially in paediatric patients with inhibitors. Emicizumab is currently the only novel marketed agent and is increasingly accessible for people with haemophilia A with and without inhibitors [4]. In many developed countries, emicizumab has become the prophylactic agent of choice for patients with persistent inhibitors against FVIII with major reported benefits [5].

Several other novel agents are in different stages of development and will probably also have a major impact on the care of haemophilia patients. A new recombinant activated human factor VII (rFVIIa) variant with four amino acid substitutions (marzeptacog alfa (activated) (MarzAA)), has increased catalytic activity and prolonged half-life which allows subcutaneous dosing [6]. Other new agents which downregulate natural anticoagulants, thereby rebalancing haemostasis, are currently under study in patients with haemophilia A and B. These agents work either by blocking tissue factor pathway inhibitor (TFPI) using subcutaneous monoclonal antibodies (concizumab, NovoNordisk; marstacimab, Pfizer) or knocking down antithrombin synthesis through a subcutaneous double-stranded small interfering RNA (Alnylam) [7]. Haemophilia B patients with inhibitors, who have been clinically the most underserved subpopulation, may benefit the most from these new agents.

Emicizumab and the other agents described above will certainly have a major impact, as described below, in the care of patients with inhibitors. This is summarized in the 10 revised principles of inhibitor management originally proposed in 2018.

## Awareness of the incidence of inhibitors and risk factors throughout life

The bispecific antibody, emicizumab, has the potential to modify the natural history of inhibitor development in some patients, especially in previously untreated patients (PUPs) treated with this agent early in life to avoid exposure to exogenous FVIII. More data shall become available about the role, safety and efficacy of emicizumab in PUPs [8–10] as well as in Previously Treated Patients (PTPs).

## Early recognition and accurate diagnosis

The importance of early recognition and accurate diagnosis of patients developing inhibitors remains of high importance, regardless of the availability of these new agents. It is recommended for patients with inhibitors to have their inhibitor titre checked regularly, at least once per year and, most importantly, before any invasive procedure. Appropriate assays for inhibitor detection and quantification should be used in patients treated with emicizumab [11].

## Optimal organization of care and communication between all stakeholders

Availability of a bispecific antibody and other novel agents will most likely impact significantly on how care of inhibitor patients is organised. As stated jointly by the European Haemophilia Consortium (EHC) and European Association for Haemophilia and Allied Disorders (EAHAD), supervision of patients on novel treatment products should be exclusively conducted in European Haemophilia Comprehensive Care Centres (EHCCCs) or European Haemophilia Treatment Centres (EHTCs), thus increasing the necessity of good communication between healthcare professionals involved. The frequency of visits of inhibitor patients to their treating specialists will probably diminish due to the less intensive treatment regimens and subcutaneous administration of the novel products. Measures should be taken to avoid negative impact of changes related to these treatment innovations on the patient-doctor relationships, in particular regular multidisciplinary follow-up by the EHCCCs with the crucial support and involvement of patients’ organisations. A major responsibility for these expert centers will also be to carefully monitor potential adverse events which may be different from those seen with rFVIIa and aPCC (such as arterial or venous thrombotic events or thrombotic microangiopathy).

## Access to haemostatic agents

While inhibitors can be eradicated in patients who have access and a positive response to immune tolerance induction therapy, the inhibitors persist in many patients. These patients standardly require regular treatment with bypassing agents, ideally administered prophylactically. The bispecific antibody and other novel agents should be readily available, when licensed, especially for patients with persistent inhibitors. Clear protocols should be put in place for the management of breakthrough bleeds, as well as for the co-administration of clotting factor concentrates or bypassing agents in case of invasive procedures [12, 13].

## Inhibitor eradication by immune tolerance induction (ITI) therapy

Inhibitor eradication by immune tolerance induction (ITI) remains the best option for patients with inhibitors [14]. Treatment with the bispecific antibody or other novel agents should be considered if ITI cannot be conducted or was not successful. Studies are currently ongoing to evaluate the impact of these new agents on ITI indications and modalities [15–17].

## Access to, and optimal preparation for, surgery and other invasive procedures

Availability of the bispecific antibody and other novel therapies currently evaluated in trials has a substantial impact on access and modalities of invasive procedures and surgery for people with inhibitors. Minor invasive procedures, e.g. dental extraction or central venous access device insertion, can be successfully performed with the bispecific antibody without FVIII and tranexamic acid, given that there is a close collaboration between the haematologist and the specialist performing the procedure. Careful planning and timing of major elective surgery is mandatory, along with well-designed protocols for co-administration of bypassing agent(s) during surgery [12;13]. The current inhibitor titre at the time of surgery should be properly determined in order to evaluate whether replacement therapy with FVIII or FIX concentrates represents an alternative to the use of bypassing agents.

## Provision of specialist nursing care

It is crucial to provide nurses with continuous and practical education about the bispecific antibody and other emerging therapies, including awareness and understanding of their respective mode of action, the precautions of use, situations requiring co-treatments with factor concentrates or bypassing agents, skills for intravenous and subcutaneous infusions …

## Provision of tailored physiotherapy care and monitoring

The use of the bispecific antibody allows inhibitor patients to engage in various types of physical activities and for those already disabled has the potential to convert them into ambulatory patients, as reported previously with aPCC given prophylactically and combined with active physiotherapy [18]. Regular musculoskeletal assessment and physical coaching by experts in physiotherapy attached to EHCCCs or EHTCS should be provided to patients switched to new agents in order to prepare them physically and mentally for this important transition. However, permanent irreversible joint damage and chronic pain remain challenges that require continued tailored physiotherapy care and monitoring in the era of new therapies.

## Access to psychosocial support

The new perspectives opened by a life with much fewer bleeds and less treatment burden require major individual mental, physical and social adaptation that could benefit from the support of psychosocial experts within the multidisciplinary team.

## Involvement in research and innovation

Clinical data on the use of the bispecific antibody and other novel products in the real life remain scarce, especially in the cohort of inhibitor patients. Thus, it is essential to collect treatment and outcome data regularly in the frame of international registries and/or pharmacovigilance programs. Further international, multicentric and collaborative studies and research initiatives exploring the impact and modalities of use of the novel therapies in patients with inhibitor should be encouraged and promoted.

## Conclusion

As described above, adoption of novel non-replacement therapies for haemophilia will have a major impact on each of the 10 principles of inhibitor management published in 2018. This amendment of the 10 principles appears as a very important advocacy tool and framework in order to promote access and proper use of these revolutionary therapies for all stakeholders actively involved in the care of patients with inhibitors.

## Data Availability

Not applicable.

## Abbreviations

aPCC: Activated prothrombin complex concentrate
EAHAD: European Association for Haemophilia and Allied Disorders
EHC: European Haemophilia Consortium
EHCCC: European Haemophilia Comprehensive Care Centre
EHTC: European Haemophilia Treatment Centre
FVIII: Factor VIII
FIX: Factor IX
ITI: Immune tolerance induction
PTP: Previously treated patient
PUP: Previously untreated patient
rFVIIa: Activated recombinant FVIIa
